# Carbohydrate-restricted Diet and High-intensity Interval Training Exercise Improve Cardio-metabolic and Inflammatory Profiles in Metabolic Syndrome: A Randomized Crossover Trial

**DOI:** 10.7759/cureus.5596

**Published:** 2019-09-08

**Authors:** Amy Gyorkos, Mark H Baker, Lauren N Miutz, Deborah A Lown, Michael A Jones, Lori D Houghton-Rahrig

**Affiliations:** 1 Preventive Medicine, Biological Sciences, Western Michigan University, Kalamazoo, USA; 2 Exercise Science, Grand Valley State University, Allendale, USA; 3 Preventive Medicine, Kinesiology, University of Calgary, Calgary, CAN; 4 Preventive Medicine, Biomedical Sciences, Grand Valley State University, Allendale, USA; 5 Physical Medicine and Rehabilitation, Western Michigan University, Kalamazoo, USA; 6 Preventive Medicine, College of Nursing, Grand Valley State University, Allendale, USA

**Keywords:** restricted carbohydrate diet, metabolic syndrome, paleolithic diet, inflammation, lipoproteins, insulin sensitivity, metabolism, ketogenic diet, high-intensity interval training

## Abstract

Introduction

One approach to slow the pandemic of obesity and chronic disease is to look to our evolutionary past for clues of the changing behaviors contributing to the emergence of 'diseases of civilization'. Modern humans have deviated from the lifestyle behaviors of our ancestors that have introduced pressures (i.e. diet and activity changes) quicker than our genetic ability to respond. This caused a 'mismatch' between our biological systems and environment, leading to 'man-made' chronic diseases.

Purpose

The purpose of the study was to investigate the effects of a short-term evolutionarily informed dietary and lifestyle intervention on inflammatory and cardio-metabolic profiles in individuals characterized as having metabolic syndrome (MetS).

Methods

Twelve subjects with MetS followed a crossover design with two, four-week interventions, including a carbohydrate (CHO)-restricted Paleolithic-based diet (CRPD; <50g CHO) with sedentary activity (CRPD-Sed) and CRPD with high-intensity interval training (CRPD-Ex), separated by a four-week washout period. The HIIT exercise consisted of 10 X 60 seconds (s) cycling intervals interspersed with 60s of active recovery three d/wk for four weeks. The effects of a diet with sedentary activity as compared to a diet with exercise on body composition, as well as the cardiovascular, inflammatory, and metabolic profiles, were assessed. A two-way analysis of variance (ANOVA) with repeated measures was performed with a post-hoc analysis using a simple effects analysis with a Bonferroni adjustment. The level of statistical significance was established a priori as p < 0.05.

Results

Compared to baselines, CRPD-Sed and CRPD-Ex improved cardio-metabolic markers, including reductions in waist adiposity (-15%, -18%), body mass (-3%, -5%), body fat % (BF%; -7%, -12%), fasting plasma glucose (GLU; -20%, -27%), triglycerides (TG; -47%, -52%), fasting insulin (-34%, -39%), insulin resistance (-35%, -46%), and increased HDL-C (+22%, +36%) and VO_2max_ (+22% and +29%), respectively. CRPD-Sed and CRPD-Ex also reduced inflammatory markers, including hsCRP (-32% and-36%), TNF-alpha (-35% and -41%), IL-6 (-29% and -40%), and ICAM-1 (-19%, -23%), respectively, when compared to baseline.

Conclusion

Adopting behaviors from our evolutionary past, including diet and exercise, shows favorable cardio-metabolic and inflammatory profiles in those individuals characterized with MetS.

## Introduction

For nearly 600-million years, the ability to store dietary energy for later times has served humans well during food-scarce periods. In our current world of highly available and hyper-palatable foods, dietary energy stores in humans have increased dramatically, resulting in 1.9 billion adults classified as overweight and, of these, 600 million as obese [1].

Obesity contributes to more deaths worldwide than underweight [1]. In addition, obesity is associated with an increased risk of chronic diseases, such as cardiovascular disease, metabolic syndrome, type 2 diabetes (T2D), nonalcoholic fatty liver disease, Alzheimer dementia, and some cancers [2]. This trend has had devastating effects on health, quality of life, and health system resources.

It has been theorized that agricultural and technological advances have introduced pressures (i.e. diet and activity changes) quicker than our genetic ability to respond, causing a mismatch between our systems and the ecosystem. One approach to slow the pandemic of obesity and chronic disease is to look to our evolutionary past for clues of the changing behaviors contributing to the emergence of "diseases of civilization" [3]. Over the past 10,000 years, we have had profound changes in feeding and activity behavior, which have left an imprint on the human genome [4].

Diet has been altered from our ancestral past, which was once dominated by anti-inflammatory alkaline foods rich in fruits, vegetables, lean meats, seafood, omega-3 fatty acids, polyphenols, fiber, and plant sterols [5]. It has been replaced largely with foods such as cereal grains, omega-6 fatty acids, and trans- and saturated fats. Today, 73% of the US food supply is composed of refined sugars, refined vegetable oils, grains, and dairy that were not present in the ancestral human diet [6]. Researchers have observed improvements in blood pressure, body fat percentage, waist circumference, fasting blood sugar, lipid and metabolic profiles, gut health, as well as lower levels of systemic inflammation and oxidative stress by returning humans to an ancestral-based Paleolithic diet [7]. A carbohydrate-restricted diet has also been shown to result in the greatest weight loss [8] and, most notably, has scored the highest rank for decreasing the risk of all-cause and cause-specific mortality, when compared to other diets with varying macro-nutrient profiles [9].

Another profound deviation from our ancestral lifestyle is the dramatic decline in physical activity patterns, with an 8% reduction from the hunter-­gatherer to the agricultural era and another 14% reduction from the agricultural era to the industrial era [10]. Exercise, particularly high-intensity interval training (HIIT), has been shown to release its own anti-inflammatory markers and improve blood lipid panels, metabolic profiles, body composition, and quality of life [11-12].

It is well-known that chronic diseases are predicated by genetic and environmental contributions. Lifestyle conditions, including exercise, diet, and smoking, can influence up to 90% of whether a disease manifests itself or is silenced [13]. Our ancestral lifestyle consisted of a diet that is higher in nutrients and lower in carbohydrates and activity patterns at varying intensities. It is, therefore, the purpose of this study to examine the effects of a short-term, carbohydrate-restricted, Paleolithic-based diet with a high-intensity exercise intervention on systemic inflammation and cardiovascular, metabolic, and body composition profiles in individuals with MetS.

## Materials and methods

Study design and subjects

**Figure 1:**
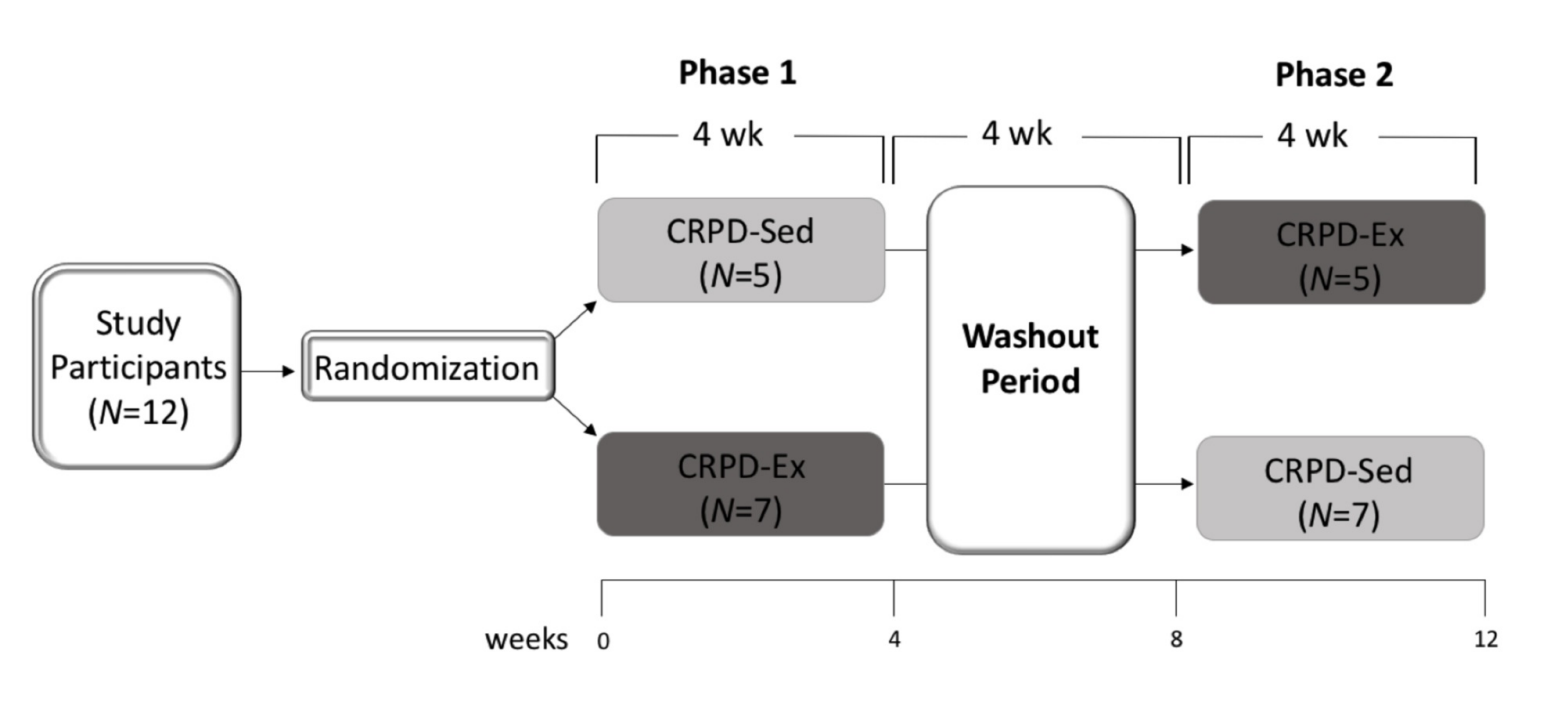
The experimental design is a randomized, two-phase crossover trial with a washout period Twelve subjects characterized by MetS completed both arms of the study, separated by a washout period. Both experimental phases and the washout period lasted four weeks each. Data collection took place at Weeks 0, 4, 8, and 12, which correlated with before and after each phase of the study. N=number of subjects randomized to that respective group; CRPD-Sed=carbohydrate-restricted Paleolithic-based diet without exercise; CRPD-Ex=carbohydrate-restricted Paleolithic-based diet with exercise.

Twelve free-living individuals completed a randomized, two-phase crossover dietary and exercise trial (Figure 1). Participants were men (n=4) and woman (n=8) between 18 and 60 years of age with Metabolic Syndrome (MetS), according to the National Cholesterol Education Program-Adult Treatment Panel III [14]. In addition to MetS, inclusion criteria were weight stable for at least three months and being relatively sedentary (defined as engaged in <30 min/day of exercise or VO2peak <45 ml.kg-1.min-1). Exclusion criteria were following any special diets and/or medications for chronic disease, and/or cardiovascular, metabolic, pulmonary, and osteoarticular disease, and associated comorbidities.

The two phases of the crossover design included: 1) carbohydrate-restricted Paleolithic-based diet with sedentary behavior (CRPD-Sed) and 2) carbohydrate-restricted Paleolithic-based diet with high-intensity interval training (CRPD-Ex). Both phases were four weeks in duration and separated by a four-week washout period, which subjects returned to baseline behaviors (Figure 1). Data collection included body composition, blood draws, and peak aerobic capacity at zero, four, eight, and 12 weeks. The study was conducted in accordance with the guidelines of the Institutional Review Board at Grand Valley State University and all participants provided written consent.

Dietary intervention and recall

Subjects were given instructions and supporting resources to follow a carbohydrate-restricted Paleolithic-based diet (CRPD). The diet consisted of unprocessed lean meat, fish, eggs, leafy and cruciferous vegetables, root vegetables, fruit, and nuts and was devoid of cereal grains, dairy, beans, legumes, refined fats, bakery items, soft drinks, beer, and extra salt and sugar. The following were recommended in limited amounts: nuts (preferentially walnuts), dried fruit, potatoes (<1 medium-sized/d), and wine (<1 glass/d). Subjects were advised to eat until full and satiated but not “stuffed” and no explicit instructions were provided regarding total caloric intake.

The goals for the macronutrient distribution of protein, fat, and carbohydrate as a percentage of total energy were 25%, 60%, and 15%, respectively. Subjects were instructed to reduce carbohydrate (CHO) consumption to <50g/d and encouraged to replace those CHO with healthy fats. Subjects were trained to use a self-monitoring phone app to improve awareness of macronutrient consumption and improve adherence to goals. The goal of the carbohydrate restriction was to induce low-level nutritional ketosis. Nutritional ketosis occurs when dietary carbohydrates are significantly reduced (<50 grams per day) and the body’s production of ketones increases to maintain a blood level at or above 0.5 mM. The primary blood ketone, β-hydroxybutyrate, was quantified using an Xtra Precision meter system (Abbott Precision Diabetes Care, Saint-Laurent, Canada) at every data collection time point (0, 4, 8, 12 wk).

A five-step, multiple-pass, 24-hour recall method [15] was used to obtain diet intake following the United States Department of Agriculture (USDA) protocol. This involved three, separate, unannounced phone calls on two weekdays and one weekend day to every participant during each arm of the study and washout period. No calls were made two days before or two days after a major holiday. The phone calls served as a mechanism of diet support and helped ensure the subject’s compliance with the diet pattern and macronutrient intake. Diet intake was analyzed for energy and nutrients using the Nutrition Data System for Research (NDSR; Minneapolis, MN). The 24-hour recalls indicated that all subjects achieved diet goals.

Exercise intervention

Participants attended three supervised exercise sessions per week for four weeks during the CRPD-Ex phase. These exercise sessions were performed privately, including one subject and one researcher. Each session was performed on a cycle ergometer and included a three-min warm-up, 10 X 60s cycling intervals interspersed with 60s of active recovery, and a three-min cool-down. Each training session was tailored to elicit ~90% maximal heart rate (HRmax) during the sprint intervals configured by the pre-trial determination of VO2peak and HRmax (see peak aerobic capacity below). Heart rate was monitored continuously and every minute of exercise recorded. Subjects pedaled slowly against resistance of 50 W during the warm-up, active recovery, and cool-down. Subjects could drink water ad libitum. All subjects tolerated and complied with the exercise protocol.

Resting heart rate and blood pressure

Resting blood pressure was taken non-invasively using an aneroid sphygmomanometer combined with a cuff containing an air bladder. The subject sat for 5 min in a comfortable environment prior to measurement. The blood pressure was recorded in even numbers to the nearest 2-mm mark on the manometer. Resting heart rate was taken by palpating the radial pulse for 30 seconds following a 5-min rest. 

Body mass and composition

All body composition measurements were taken in the morning after an overnight fast at approximately the same time of day at Weeks 0, 4, 8, and 12 of study. Body mass was measured to the nearest 100 g on a calibrated digital scale and body mass index (BMI) was calculated (kg/m^2^). Circumference measurements were taken by the same technician using standard procedures with a 150-cm anthropometric spring-loaded measuring tape. Waist circumference was measured at the narrowest part of the torso, level with the “natural” waist between the ribs and iliac crest and measured at the end of normal expiration. The total body density was indirectly measured via the underwater weighing technique according to Eston and Reilly [16] and the Brozek formula was used to determine fat mass (kg) and percent body fat.

Peak aerobic capacity

A maximal continuous graded exercise test (GXT) on a cycle ergometer was completed by all participants to determine peak oxygen consumption (VO2peak) and maximum heart rate (HRmax). Participants began pedaling at a cadence of 60-80 revolutions per minute (RPM) at a light exercise intensity of ~60 watts (W). The workload increased 20W every minute until the subject reached volitional fatigue or could no longer maintain the required power output (60-80 RPM), despite verbal encouragement. Respiratory gases were monitored continuously and analyzed with open-circuit spirometry using a calibrated metabolic cart (True One 2400, Parvo-Medics, Inc., Provo, UT). During the final 30s of each minute of the GXT, participants reported their overall ratings of perceived exertion (RPE) using the Borg 6-20 RPE scale.

All subjects reached volitional fatigue within 8-15 min of exercise. In conjunction with volitional fatigue, the maximal effort was confirmed with two of the following criteria: (a) heart rate (HR) within 10% age-predicted HRmax, (b) plateau in VO2, (c) respiratory exchange ratio of greater than 1.15, and/or (d) RPE of ≥18 [17]. Data were averaged over 15-second intervals with the highest VO2 and HR recorded as VO2peak and HRmax, respectively.

Blood analysis

Fasting whole blood was collected via the aseptic technique from the antecubital vein at Weeks 0, 4, 8, and 12 of study at least 72 hours post-exercise. Blood was collected in tubes (BD Vacutainer® plus SST; Becton Dickinson, NJ, US), centrifuged at 1.500 x g for 15 min and aliquoted into separate storage tubes. Total cholesterol (TC), high-density lipoprotein (HDL), triglycerides (TG), and glucose (GLU) were immediately analyzed and quantified via reflectance photometry with the Cholestech LDX® system (Alere, UK). Low-density lipoprotein (LDL) was calculated using the Friedewald formula as follows: (LCL-C=TC-HDL-C (TG/5)). High-sensitivity C-reactive protein (hsCRP) was detected and quantified by a turbidimetric immunoassay reagent kit (Catalog# DZ135A-K) and an automated clinical analyzer (SMART®, Diazyme Laboratories, Poway, CA).

The remaining serum was stored frozen at -80°C and thawed only once before analysis. Commercial enzyme-linked immunosorbent assays (ELISAs) were used for the detection and quantification of insulin, tumor necrosis factor-alpha (TNF-α), interleukin-6 (IL-6), and intercellular adhesion molecule-1 (ICAM-1). The commercial reagents kits were all purchased from Thermo Fisher Scientific (#catalog) and sensitivity and intra-assay coefficient of variation (CV) followed; insulin (#KAQ1251) was 0.28 µUI/ml, 5.8%, TNF-α (#EH3TNFA2) was 5.6 ng/ml, 4.7%, IL-6 (#EH2IL62) was 1.1 ng/ml, 2.9%, and ICAM-1 (#EHICAM1) was 0.156ng/ml, 6.1%. The 96-well plates were read with an absorbance microplate reader (BioTek Instruments Inc., VT, US). Insulin sensitivity was calculated with the homeostatic model assessment of insulin resistance (HOMA-IR)=(fasting glucose × fasting insulin)/22.5.

Statistical analysis

Data were analyzed using a two-way analysis of variance (ANOVA) with repeated measures to evaluate changes over time (0, 4, 8, 12 wks) and condition (CRPD-Sed, CRPD-Ex) for all variables. Post-hoc analysis of the data was performed using Tukey’s procedure for simple effects analysis with Bonferroni adjustment. The level of statistical significance was established a priori as P < 0.05. A statistical power analysis was determined (GPower software; Universität Düsseldorf) by a priori F test, specifically a within-­subject ANOVA with repeated measures statistical test (0.5 effect size, 0.05 alpha err, 0.8 power). The results indicated a total sample size of 12. All statistical analysis was performed using SPSS 24 software (IBM Corp., Armonk, NY, US). The level of statistical significance was established a priori as p < 0.05. Values are reported as means ± standard deviation (SD).

## Results

Dietary intake

The average nutrient intake is provided in Table 1. Although the subjects were not counseled to restrict calories, a spontaneous reduction in total caloric intake was observed when comparing baseline to CRPD-Sed (2189 ± 689 to 1306 ± 539 kcal; p ≤ 0.05) and baseline to CRPD-Ex (2466 ± 602 to 1590 ± 587 kcal; p ≤ 0.05). This effect is consistent with other carbohydrate-restricted diet studies [18]. The diet interventions significantly altered nutrient composition during the CRPD-Sed as compared to baseline (1306 kcal; %CHO:fat:protein = 16:62:22 vs 2189 kcal; 49:37:15) and CRPD-Ex as compared to baseline (1590 kcal: %CHO:fat:protein = 13:67:22 vs 2466 kcal; 45:43:13). The diet composition results were in line with diet instructions and study objectives, indicating high dietary compliance. A significant increase in saturated fats, monounsaturated fats, and cholesterol was observed following CRPD intervention when compared to baseline and washout. There was no significant change between diet intervention phases (CRPD-Sed and CRPD-Ex) nor between baselines. Blood ketone β-hydroxybutyrate averaged 0.53 ± 0.19 mmol/L following the diet intervention phases, also indicating a high degree of dietary compliance.

**Table 1:** Average nutrient intake for subjects during baseline and diet interventions Values are mean ± SD; (*) denotes significance between baseline and Week 4 of intervention at p ≤ 0.05; CRPD-Sed=carbohydrate-restricted Paleolithic-based diet with sedentary (without exercise); CRPD-Ex=carbohydrate-restricted Paleolithic-based diet with exercise

Variable	CRPD-Sed Baseline	Post 4wks	CRPD-Ex Baseline	Post 4 Wks
Energy (kcal)	2189 ± 689	1306 ± 539*	2466 ± 602	1590 ± 587*
Protein (g)	82 ± 32	71 ± 39	80 ± 36	87 ± 30
Protein (% energy)	15 ± 7	22 ± 6*	13 ± 5	22 ± 7*
Carbohydrate (g)	268 ± 98	52 ± 9*	277 ± 105	51 ± 7*
Carbohydrate (% energy)	49 ± 6	16 ± 5*	45 ± 9	13 ± 4*
Total Fat (g)	202 ± 39	90 ± 35*	117 ± 31	118 ± 27
Total Fat (% energy)	37 ± 9	62 ± 13*	43 ± 11	67 ± 11*
Saturated Fat (g)	37 ± 12	43 ± 17*	34 ± 11	40 ± 14*
Monounsaturated Fat (g)	22 ± 7	38 ± 12*	24 ± 9	42 ± 17*
Polyunsaturated Fat (g)	12 ± 4	14 ± 6	12 ± 6	17 ± 9*
Alcohol (% energy)	2 ± 2	1 ± 2	1 ± 1	1 ± 1
Cholesterol (mg)	443 ± 189	654 ± 289*	398 ± 162	687 ± 279*

CRPD-Sed and CRPD-Ex improve MetS risk factors in MetS

The study sample included eight women and four men (N=12) with a mean age of 40.9 ± 20.2 characterized with MetS by elevated levels of adiposity (115 ± 7.4 cm), SBP (130 ± 9.7 mm/Hg), HDL-C (32 ± 5.2 mg/dL), TG (216 ± 35.5 mg/dL), and fasting glucose (114 ± 7.2 mg/dL). All MetS risk factors significantly improved following both CRPD-Sed and CRPD-Ex, including waist adiposity (-15%, -18%; Table 2), SBP (-5%, -5%; Table 4), HDL (+22%, +36%; Table 3), TG (-47%, -52%; Table 3), and fasting glucose (-20%, -27%; Table 4), respectively, when compared to baseline. MetS risk factors improved both with CRPD-Sed and CRPD-Ex from the usual diet. However, CRPD-Ex resulted in significantly greater improvements in waist adiposity, HDL, TG, and fasting glucose as compared to CRPD-Sed.

**Table 2:** Change in body composition following restrictive carbohydrate diet with and without exercise Values are mean ± SD; *Significance between baseline and week 4 of intervention at p ≤ 0.05; † Significance between CRPD-Sed and CRPD-Ex at p ≤ 0.05; ‡ Significance between CRPD-Sed and CRPD-Ex at p ≤ 0.02 BMI=body mass index; CRPD-Sed=carbohydrate-restricted Paleolithic-based diet with sedentary (without exercise); CRPD-Ex=carbohydrate-restricted Paleolithic-based diet with exercise

	CRPD-Sed		CRPD-Ex	
Variable	Baseline	Post 4 wks	Baseline	Post 4 wks
Body Mass (kg)	97.2 ± 16.7	94.1 ± 12.1*	97.6 ± 15.9	92.4 ± 12.2*^†^
BMI (kg/m^2^)	35.4 ± 4.3	33.2 ± 5.4*	34.9 ± 6.3	30.0 ± 3.4*^†^
Body fat (%)	42.8 ± 6.4	39.6 ± 7.6*	42.5 ± 5.9	37.2 ± 6.2*^†^
Waist Adiposity (cm)	115 ± 7.4	98.0 ± 10.2*	111.4 ± 5.8	91 ± 9.1*^‡^

**Table 3:** Change in lipid profile following restrictive carbohydrate diet with and without exercise Values are mean ± SD; *Significance between baseline and Week 4 of intervention at p ≤ 0.05; † Significance between CRPD-Sed and CRPD-Ex at p ≤ 0.05; ‡ Significance between CRPD-Sed and CRPD-Ex at p ≤ 0.02 TC=Total Cholesterol; HDL-C=High Density Lipoprotein-Cholesterol; LDL-C=Low-Density Lipoprotein-Cholesterol; TG=Triglyceride; CRPD-Sed=carbohydrate-restricted Paleolithic-based diet with sedentary (without exercise); CRPD-Ex=carbohydrate-restricted Paleolithic-based diet with exercise

	CRPD-Sed		CRPD-Ex	
Variable	Baseline	Post 4 wks	Baseline	Post 4 wks
TC (mg/dL)	222 ± 21.5	219 ± 32.1	225 ± 26.5	209 ± 21.8*^‡^
HDL-C (mg/dL)	32 ± 5.2	39 ± 7.7*	33 ± 4.3	45 ± 10.1*^‡^
LDL-C (mg/dL)	129 ± 23.5	135 ± 24.5	132 ± 26.9	130 ± 21.7
TG (mg/dL)	216 ± 35.5	114 ± 32.9*	196 ± 31.1	94 ± 25.6*^‡^
TC/HDL-C Ratio	6.2 ± 1.1	2.9 ± 0.7*	6.0 ± 1.3	2.3 ± 0.9*^†^

**Table 4:** Change in cardio-metabolic profile following restrictive carbohydrate diet with and without exercise Values are mean ± SD; *Significance between baseline and Week 4 of intervention at p ≤ 0.05; † Significance between CRPD-Sed and CRPD-Ex at p ≤ 0.05; ‡ Significance between CRPD-Sed and CRPD-Ex at P≤ 0.02 SBP=Systolic Blood Pressure; GLU=Fasting Glucose; HOMA-IR=homeostasis model assessment-insulin resistance; VO2peak=peak aerobic capacity; CRPD-Sed=carbohydrate-restricted Paleolithic-based diet with sedentary (without exercise); CRPD-Ex=carbohydrate-restricted Paleolithic-based diet with exercise

	CRPD-Sed		CRPD-Ex	
Variable	Baseline	Post 4 wks	Baseline	Post 4 wks
SBP (mm/Hg)	130 ± 9.7	124 ± 6.6*	128 ± 9.4	122 ± 7.6*
GLU (mg/dL)	114 ± 7.2	91 ± 8.4*	111 ± 10.2	81.3 ± 9.1*^‡^
Fasting Insulin (µUI/ml)	19.3 ± 9.6	12.8 ± 9.1*	18.2 ± 8.7	11.3 ± 9.4*
HOMA-IR	2.6 ± 1.8	1.7 ± 0.9*	2.4 ± 1.1	1.3 ± 1.2*^†^
VO_2peak_(mL·kg^-1^·min^-1^)	23 ± 7.4	28 ± 5.5*	24 ± 8.2	31 ± 6.1*^†^

CRPD-Sed and CRPD-Ex improve body composition in MetS

Both CRPD-Sed and CRPD-Ex resulted in a significant improvement in body composition, including a reduction in body mass (-3%, -5%), BMI (-6%, -14), %body fat (-7%, -12%), waist adiposity (-15%, -18%), respectively, when compared to baseline (Table 2). The addition of HIIT exercise with CRPD significantly improved all body composition markers above those observed following sedentary behavior (Table 2).

CRPD-Sed and CRPD-Ex improve select lipid markers in MetS

CRPD-Sed and CRPD-Ex interventions significantly improved select lipid markers, including HDL (+22% vs +36%), TG (-47% vs -52%), and TC/HDL-C (-53% vs 62%), respectively, when compared to baseline (Table 3). CRPD-Ex significantly improved these markers above those observed following CRPD-Sed. In addition, CRPD-Ex significantly reduced TC (-7%) when compared to both baseline and CRPD-Sed. LDL-C remained unaltered following either intervention (Table 3).

CRPD-Sed and CRPD-Ex improve cardio-metabolic profile in MetS

At baseline, all subjects had a metabolic milieu associated with dysregulated cardio-metabolic profiles (Table 4). CRPD-Sed and CRPD-Ex result in improvements of all measured metabolic markers, including reductions in SBP (-5%, -5%), glucose (-20%, -27%), insulin (-34%, -39%), and HOMA-IR (-35%, -46%) levels while increasing peak aerobic capacity; VO2peak (+22%, +29%), respectively, when compared to baseline. CRPD-Ex significantly improved select metabolic markers above those found following CRPD-Sed, including fasting glucose, insulin resistance, and VO2peak (Table 4).

CRPD-Sed and CRPD-Ex improve the inflammatory profile in MetS

CRPD, independently of exercise level, significantly improved all measured inflammatory markers over the four-week intervention (Table 5). CRPD-Sed and CRPD-Ex significantly decreased high-sensitivity C-reactive protein (hsCRP) (-32%, -36%), tumor necrosis factor-alpha (TNF-α) (-35%, -41%), interleukin-6 (IL-6) (-29%, -40%), and intercellular adhesion molecule 1 (ICAM-1) (-19%, -23%), respectively, when compared to baseline. No significant changes were observed between CPRD-Sed and CPRD-Ex interventions.

**Table 5:** Change in inflammatory profile following restrictive carbohydrate diet with and without exercise Values are mean ± SD; *Significance between baseline and Week 4 of intervention at p ≤ 0.05; hsCRP=high sensitivity C-reactive protein; TNF-α=tumor necrosis factor-alpha; IL-6=interleukin-6; ICAM-1=intercellular adhesion molecule 1; CRPD-Sed=carbohydrate-restricted Paleolithic-based diet with sedentary (without exercise); CRPD-Ex=carbohydrate-restricted Paleolithic-based diet with exercise

	CRPD-Sed		CRPD-Ex	
Variable	Baseline	Post 4 wks	Baseline	Post 4 wks
hsCRP (pg/mL)	4.1 ± 1.9	2.8 ± 1.4*	3.9 ± 1.7	2.5 ± 1.4*
TNF-α (pg/ml)	3.6 ± 1.3	2.3 ± 0.6*	3.2 ± 1.5	1.9 ± 0.4*
IL-6 (pg/ml)	3.8 ± 1.2	2.7 ± 0.8*	3.5 ± 0.9	2.1 ± 0.6*
ICAM-1 (ng/ml)	296 ± 107	239 ± 78*	289 ± 110	223 ± 98*

## Discussion

In the current study, CRPD, regardless of activity level, improved body composition, cardio-metabolic profiles, and inflammatory profiles in individuals with metabolic syndrome after a four-week intervention. The addition of HIIT exercise to the diet further improved body composition and cardio-metabolic profiles when compared to diet alone.

Improvements in body composition

The current study resulted in CRPD with and without HIIT exercise significantly improving body composition, including body mass, % body fat, and waist circumference when compared to baseline. These findings are in agreement with a strong body of evidence that carbohydrate restriction stimulates weight loss and reduces waist adiposity as well or better than other diets [18]. Several theories have been proposed to explain the carbohydrate-restricted induced fat loss mechanism, including increased satiety with reductions in appetite, reductions in lipogenesis and increased lipolysis, increased metabolic costs of gluconeogenesis, and greater metabolic efficiency in consuming fats [19].

We are the first to demonstrate that the impact of CRPD on body composition is enhanced by HIIT. Previous studies have only investigated either HIIT or diet alone. The addition of HIIT exercise to CRPD improved body composition above that observed following a diet alone. We did not examine the effects of exercise alone, but others have shown that in short-term high-intensity exercise interventions (2-6 weeks), negligible fat loss is achievable [20]. Although speculative, given the results from other studies, the rapid short-term body composition changes observed following CRPD-Ex was not likely a result from exercise alone but the combination of carbohydrate-restricted diet and HIIT exercise. Therefore, we demonstrated that CRPD with sedentary activity resulted in improvement in body composition, with a greater improvement occurring with diet and HIIT.

Improvements in inflammatory profile

In the current study, CRPD with sedentary activity and HIIT improved the inflammatory profile in individuals with metabolic syndrome after a four-week intervention. The addition of HIIT exercise to the diet did not improve inflammatory markers further when compared to CRPD-Sed. This is consistent with studies that show low carbohydrate ancestral-based diets that are more nutrient-dense, lower in ω-6 to ω-3 fatty acid ratio and glycemic load, higher in fiber intake and antioxidant capacity, and more alkaline are associated with lower inflammatory profiles [21].

One possible explanation for the improvement in inflammation following the diet intervention is the parallel improvement in body composition described by others. Sharman & Volek (2004) showed that weight loss improves inflammation following both very-low-carbohydrate and energy-restricted low-fat diets [22]. It is well-established that adipose tissue is an endocrine organ capable of releasing its own adipocytes, including tumor necrosis factor (TNF)-α, interleukin (IL)-6, leptin, adiponectin, and others and is a key plexus in the development of chronic metabolic diseases, impairing the healthy immune-metabolic crosstalk [23].

Using this theory, we would have expected that the CRPD-Ex intervention that resulted in greater improvements in body composition would have also led to greater improvements in the inflammation profile when compared to CRPD-Sed, but no statistical significance was evident. An alternative theory is that the macronutrient composition of the diet significantly altered the inflammatory profile independent of weight loss as shown with other studies. Forsythe et al. (2007) showed that when comparing two diets of isocaloric intake and similar weight loss, the very-low-carbohydrate-ketogenic diet had more significant reductions in select pro-inflammatory markers than the low-fat diet [24].

Improvements in the lipoprotein profile

Lipoprotein Profile

Carbohydrate-restricted diets have been shown to consistently improve lipid and metabolic profiles in healthy, overweight, and obese individuals when compared to the baseline and low-fat diets. In the current study, CRPD increased HDL-C levels, reduced TG and TC/HDL-C ratio, and had no effect on LDL cholesterol. These findings are consistent with other short- and long-term studies, including the longest study to date by Shai and colleagues [25]. The researchers showed that a low carbohydrate diet improved HDL, TG, TC/HDL levels significantly and steadily over a two-year span when compared to a low-fat diet. The lipid findings should be interpreted with caution as short-term changes are influenced by weight change [26]. Researchers have consistently shown, however, that regardless of weight change, more significant lipid results follow a low carbohydrate diet when compared to low-fat diets [19,27].

The current study extends existing data by examining the effects of complementing the carbohydrate-restricted diet with high-intensity interval training. The addition of HIIT exercise significantly improved the TC, HDL-C, TG, and TC/HDL-C ratio when compared to the baseline and CRPD-Sed. HIIT has previously been reported to raise HDL-C, lower TG, and reduce oxidized low-density lipoproteins when compared to the baseline and moderate-intensity continuous training [28]. We show here that by combining a restricted carbohydrate diet with HIIT exercise, the overall lipid panel was significantly improved when compared to the baseline and CRPD-Sed.

Improvements in the cardio-metabolic profile

Metabolic Profile

This study realized a further reduction in fasting glucose and improved insulin sensitivity when combining diet and exercise intervention when compared to diet alone. These findings are consistent with studies showing HIIT exercise is effective in improving insulin sensitivity, glycemic control, and diabetes-related outcomes [28]. Further studies are needed in order to tease out the effects of combined diet and exercise versus exercise alone on glycemic regulation and insulin resistance. A carbohydrate-restricted diet is a logical intervention for those suffering from hyperglycemia and hyperinsulinemia and has consistently shown remarkable reductions in fasting and postprandial glucose and insulin levels.

Cardiorespiratory Fitness Profile

The current study also examined the effects of carbohydrate restriction with and without HIIT exercise on cardiorespiratory fitness levels (VO2peak). Although often overlooked, an individual’s cardiorespiratory fitness level is a significant health risk factor, as fitness has been found to be inversely associated with metabolic syndrome incidence and is a chief predictor of cardiovascular disease and all-cause mortality [29].

CRPD-Sed was able to improve peak aerobic capacity by 3.2 mL·kg-1·min-1 and CRPB-Ex by 7.4 mL·kg-1·min-1 when compared to the baseline. According to Blair and colleagues, a reduction of 1.44 mL·kg-1·min-1 in VO2peak corresponds to a 7.9% reduction in overall mortality [29]. Applying that conversion here, subject’s risk of all-cause mortality was reduced to 17.5% and 41% following CRPB-Sed and CRPB-Ex, respectively. The rise in fitness level following CRPD-Ex was more exaggerated than the results reported from combining a Paleolithic-based diet with moderate aerobic exercise in type 2 diabetics [30]. This may be due to the different intensities of exercise as HIIT alone has been shown to alter VO2peak by nearly twofold when compared to moderate-intensity continuous training [11]. The investigated underlying mechanisms for HIIT advantage have been summarized elsewhere [11].

Limitations

The individual arms of this crossover study lasted four weeks each and included 12 subjects. This can be viewed as small sample size and short in duration as compared to larger clinical trials. The benefits of conducting such a study offer preliminary results and insight for future larger studies. Further, the small size increases the integrity of the study with high levels of attrition, compliance, and experimental control for subjects that contributed to statistical significance being achieved on most variables. This short-duration study may be clinically meaningful as a reversal of metabolic syndrome occurred in only one month. Future research is needed to further elucidate these findings with a larger sample size over a longer period of time.

## Conclusions

In summary, this study supports that carbohydrate-restricted Paleolithic-based diets can reverse metabolic syndrome by improving body composition and the inflammatory and cardio-metabolic profiles. To our knowledge, this is the first study that has examined the effects of supplementing this diet with HIIT exercise in those characterized with MetS. We have shown that the addition of exercise can further improve body composition, lipoprotein profile, insulin sensitivity, and aerobic performance beyond the results of diet alone.
